# Diabetes Mellitus Type 2 and Popular Misconceptions: A Cross‐Sectional Study

**DOI:** 10.1002/hsr2.71308

**Published:** 2025-10-03

**Authors:** Anahita Babak, Shiva Rouzbahani, Alireza Safaeian, Farzam Poonaki

**Affiliations:** ^1^ Department of Community and Family Medicine, School of Medicine Isfahan University of Medical Sciences Isfahan Iran; ^2^ Miller School of Medicine, Bascom Palmer Eye Institute University of Miami Miami Florida USA; ^3^ Department of Community Medicine and Family Physician, School of Medicine Isfahan University of Medical Sciences Isfahan Iran; ^4^ School of Medicine Isfahan University of Medical Sciences Isfahan Iran

**Keywords:** diabetes management, diet, misconceptions, patient education, type 2 diabetes mellitus

## Abstract

**Background and Aims:**

Diabetes mellitus is a chronic condition demanding continuous monitoring to mitigate potential complications. One major obstacle to effective treatment lies in widespread misconceptions across key domains, including pathogenesis, nutrition and lifestyle, management and follow‐up, and complications and social stigma. This study aimed to evaluate patient misconceptions about the causes and management of diabetes and explore their prevalence concerning factors such as age and gender.

**Methods:**

This cross‐sectional study was conducted in Isfahan, Iran, between 2021 and 2022. A total of 390 individuals diagnosed with type 2 diabetes mellitus were included through a convenient sampling method from different diabetes centers. Data were collected using a questionnaire about the causes of diabetes mellitus, dietary guidelines, medical interventions, and associated complications. The results were analyzed with a *χ* ^2^ test using SPSS software v. 16.

**Results:**

A total of 390 participants, with a mean age of 56 ± 13.4 years and 58% females, participated in the study. The most prevalent myths about diabetes mellitus identified in this study include: only solid oils are fattening (302, 77.4%), diabetic patients are permitted to take any quantity of dried mulberries (201, 51.5%), diabetes is only a hereditary disease (177, 45.3%), diabetes can only strike obese individuals (167, 42.8%), and fruits can be consumed in any quantity by diabetic individuals because fruit sugar is a natural substance (143, 36.6%).

**Conclusion:**

Patients' misunderstandings of the etiology and management of diabetes mellitus are diverse. Given that myths can negatively affect glycemic control; patient education is crucial to effective management.

## 1 Introduction

Diabetes mellitus (DM) is a life‐long condition affecting millions globally. The International Diabetes Federation has estimated that DM has affected ~10.5% of the adult population (half a billion), and this number is expected to increase by 46% by the Year 2045, affecting the lives of over 700 million people [1]. Iran is no exception to these circumstances; 8.7% of Iran's 15–64 years population is struggling with DM, and incidence rates are increasing rapidly [2, 3]. With type 2 diabetes (T2D) accounting for more than 90% of diabetes cases, it is crucial to identify factors that contribute to disease prognoses, such as patient mindset and misconceptions around diabetes causes, treatments, side effects, prevention, and alleviating and aggravating factors [4, 5].

Chronic side effects of DM pose a serious threat to the affected individuals as well as the healthcare system in terms of economic burden. Appropriate glycemic control is the cornerstone of disease management, which relies on sustained long‐term self‐management [6]. Patient misinformation and misconceptions can be a barrier to effective treatment, increasing the patient's likelihood of DM‐related consequences [7, 8].

These false beliefs vary across populations and cultures. While some of them, such as “when blood sugar is high, I feel it” and “when blood sugar is normal, there is no need to take medicine,” were similar in some societies, there were also different misconceptions among them [9, 10, 11]. For instance, a recent study showed that the most common misconceptions among patients with DM in Eastern Saudi Arabia were “Being overweight causes diabetes” and “Diabetes is only a hereditary disease” with a high percentage of 89 and 80, respectively [9]. On the other hand, common misconceptions in Taiwan were “taking insulin hurts the kidneys and may result in a need for dialysis” and “being a vegetarian helps control blood sugar levels” by around half of the respondents [10]. In addition, over half of the patients from low‐income minorities in the United States (56%) believed that normal glucose level is no more than 200 mg/dL [11].

A study conducted in the south of Iran on patients with T2D requiring insulin revealed that around 60% of them were disinclined to insulin use due to several factors, including misconceptions or irrational fear toward this therapeutic approach [12]. Torabian et al. [13] and Ghadiri‐Anari et al. [14] have also pointed out similar misconceptions about insulin use and the importance of these misconceptions on blood sugar control in their studies.

Although a thorough understanding of the existing factors is essential for initiating effective patient education, to the best of our knowledge, no comprehensive study has examined the beliefs of diabetes patients in the Iranian population across all dimensions. Moreover, prior studies [12, 13, 14] have primarily focused on specific groups, such as insulin or oral medication users, rather than addressing diabetes more broadly. Compared to similar studies conducted in Iran and other countries, our study adopts a more comprehensive approach. Unlike previous research that primarily used yes/no questions or focused on specific aspects of diabetes, we categorized misconceptions more thoroughly and included open‐ended questions. This study aims to assess common misconceptions that contribute to poor disease management in diabetes patients, regardless of their treatment type. Additionally, it explores the prevalence of these misconceptions concerning factors such as age and gender as a secondary objective.

## 2 Materials and Methods

### 2.1 Study Design and Participants

This cross‐sectional study was conducted between 2021 and 2022 at multiple healthcare facilities affiliated with Isfahan University of Medical Sciences (IUMS), Iran. The study protocol was reviewed and approved by the Research Ethics Committee of IUMS (Ethics Code: IR.MUI.MED.REC.1401.276), and all procedures were conducted in accordance with the Declaration of Helsinki. Written informed consent was obtained from all participants prior to enrollment.

#### 2.1.1 Sample Size Calculation

Isfahan has an estimated population of 2,200,000, with a reported diabetes prevalence rate of ~10% [15]. Using Cochran's formula (Morgan's table) and assuming a 50% prevalence of misconceptions to maximize the required sample size, a confidence level of 95%, and a statistical power of 80%, the minimum sample size was calculated to be 384 participants. To accommodate potential exclusions or nonresponses, the target recruitment was set at 390 patients with type 2 diabetes mellitus (T2DM).

#### 2.1.2 Sampling Methodology

Due to the absence of a comprehensive patient registry or a feasible random sampling framework, a two‐stage, nonprobability sampling strategy was adopted to enhance generalizability while maintaining logistical practicality. In the first stage, nine healthcare centers were purposively selected to reflect a wide range of patient demographics, socioeconomic backgrounds, and levels of healthcare access. These included four public primary care health centers, two private diabetes clinics, one university‐affiliated referral hospital, one charity outpatient clinic, and one endocrinology and metabolism research center. This diverse selection was intended to ensure adequate representation from across the diabetic population of Isfahan.

In the second stage, participants were selected through consecutive convenience sampling within each facility. Adults aged 18 years or older with a confirmed diagnosis of T2DM for at least 6 months, who could communicate in Persian (Farsi), and who were presenting for routine care were considered eligible. Patients with gestational diabetes, those experiencing acute medical illness requiring urgent care, or those with significant cognitive impairment that might interfere with consent or survey completion were excluded.

Trained interviewers were stationed at each facility, with one interviewer assigned per site during scheduled visit days. On each visit, interviewers screened patient rosters and approached eligible individuals consecutively based on their order of arrival. After verifying eligibility and obtaining consent, interviews were conducted face‐to‐face in private settings. Each center was visited on three to five separate weekdays, including both morning and afternoon clinic sessions, to minimize time‐based selection bias.

To promote balance across centers, interviewer time was allocated proportionally to the average weekly patient volume at each site, and no single center contributed more than 15% to the final sample. While the sampling strategy was nonrandom by design, it was optimized to capture diversity and reduce bias within the constraints of a real‐world outpatient setting.

### 2.2 Questionnaire Design

Misconceptions related to diabetes were initially extracted from a previous quantitative study [16] and supplemented with a thorough literature review. Based on this information, a checklist of Yes/No items was compiled to identify common misconceptions. The questionnaire was developed, pilot‐tested, and distributed in Persian (Farsi), the native language of the participants, to ensure clarity and cultural relevance.

To further refine the questionnaire, extensive discussions were held with a multidisciplinary team of diabetes care professionals, including an endocrinology faculty member, two endocrinologists, one resident specializing in endocrinology and metabolism, two internists, two general practitioners, one nursing expert, one senior nutritionist, and one health expert. Their feedback, along with the patient beliefs they had observed in clinical practice, was used to shape the final version of the questionnaire, which consisted of 21 items, including both multiple‐choice and open‐ended questions.

The preliminary questionnaire was then pilot‐tested on 20 eligible participants to assess clarity and relevance, and modifications were made as necessary. To ensure its reliability, internal consistency was measured using Cronbach's *α*, resulting in a coefficient of 0.85, while test–retest reliability was confirmed with Pearson's correlation coefficient, yielding a value of 0.58.

### 2.3 Final Survey

The questionnaire encompassed various sections, including demographic characteristics (such as gender, age, education, place of reference, duration of diabetes, and type of treatment), three multiple‐choice questions related to diabetes etiology, six multiple‐choice questions, and two open‐ended questions on dietary information and lifestyle, six multiple‐choice questions and two open‐ended questions concerning diabetes management and follow‐up, and one multiple‐choice question along with one open‐ended question about diabetes complications. To avoid misinforming participants by using only misconceptions or nonfactual information, the questionnaire was designed to include a balanced mix of factual, nonfactual, and counterfactual statements. This ensured that participants were not led to form incorrect assumptions and allowed us to accurately capture their existing knowledge and beliefs about diabetes. Open‐ended questions addressed diabetes complications, the number of meals and snacks consumed per day, and, if applicable, reasons for concern about insulin use. In these open‐ended questions, participants provided their responses, and scientifically incorrect answers were considered misconceptions and grouped together based on similarity. In contrast, for multiple‐choice questions, participants were allowed to select more than one item. For example, in response to the question “What are the causes of diabetes?”, they could choose any options they deemed appropriate from the ones provided. If they identified another reason not listed among the available options, they could include it in the “other reasons” section. Similarly, false choices were categorized as misconceptions.

### 2.4 Statistical Analysis

The data were analyzed using Statistical Package for the Social Sciences (SPSS) version 16 for Windows (SPSS Inc., Chicago, IL). All data were categorical and reported as frequency (percentage). The *χ* ^2^ was utilized to evaluate the association between misconceptions and demographic variables such as age group and gender. The degrees of freedom (df) for each *χ* ^2^ test were reported, indicating the number of independent values or quantities that can vary in the statistical calculations, which depends on the number of categories involved in the test. Effect sizes were calculated using Phi (Φ) for 2 × 2 comparisons and Cramer's *V* for larger contingency tables to quantify the strength of the associations. A *p* value below 0.05 was considered statistically significant, and all tests were two‐sided.

## 3 Results

### 3.1 General Data

A total of 390 patients with a mean age of 56 ± 13.4 years completed the survey. As the questionnaires were conducted through interviews, there were no incomplete items. Over 58% (229) of the participants were females. The age groups were distributed into four subgroups: young adult (18–25), adult (26–44), middle‐aged (45–59), and old age (60+) with the highest frequency in the middle‐aged (40.8%). In total, 110 participants (28%) had a college‐level education. The median (interquartile range) of DM duration was 7 (9) years, and 262 participants (67.1%) were only taking oral medication. Table 1 demonstrates the participants' demographic data and Table 2 demonstrates the most frequent misconceptions in general and also by gender.

**Table 1:** Demographic data of the participants.

Demographic parameters	Total (390)
Gender	
Male	41.3% (161)
Female	58.7% (229)
Age groups	
Young adult	1.3% (5)
Adult	17.4% (68)
Middle age	40.8% (159)
Old age	40.5% (158)
Education	
Illiterate	15.4% (60)
Primary	20.3% (79)
High school	11.3% (44)
High school graduate	24.6% (96)
College	28.5% (111)
Health centers	
Private diabetes clinics	54.4 (212)
Endocrinology and Metabolism Research Center	16.4% (64)
Charity clinics	10.5% (41)
University‐affiliated referral hospital (Endocrinology Department)	10% (39)
Public health centers	8.5% (33)
Treatment type	
Oral	67.2% (262)
Insulin	13.8% (54)
Oral and insulin	12.6% (49)
Untreated	6.4% (25)

**Table 2:** Frequency (percentage) of the most common misconceptions across different fields, stratified by gender.

Misconception	Female (229)	Male (161)	Total (390)
**Field 1 – Pathogenesis misconceptions**			
Diabetes is only a hereditary disease	114 (49.78%)	63 (39.1)	177 (45.3%)
Only obese people can get diabetes.	88 (38.4%)	79 (49.1%)	167 (42.8%)
Diabetes only occurs in old age.	2 (0.9%)	2 (1.2%)	4 (1%)
Diabetes is a contagious disease.	3 (1.3%)	2 (1.2)	5 (0.1%)
Stress is the primary cause of diabetes.			16 (4.1%)
**Field 2 – Nutritional and lifestyle misconceptions**			
2‐1. Main meals and snacks			
A patient with diabetes requires a daily intake of more/less than three main meals.	37 (16.1%)	32 (19.8%)	69 (17.6%)
Patients with diabetes must only take less than two snacks a day.	20 (12.4%)	15 (6.5%)	35 (8.9%)
In patients with diabetes, only one light snack per day is enough.			32 (8%)
2‐2. Special foods			
Patients with diabetes should completely stop eating pasta.	76 (33.2%)	49 (30.4%)	125 (32%)
Patients with diabetes should completely stop eating potatoes.	57 (24.9%)	31 (19.3)	88 (22.5%)
Patients with diabetes should completely stop eating rice.	47 (20.5%)	31 (19.3%)	78 (20%)
Patients with diabetes should completely stop eating bread (homemade or otherwise).	46 (20%)	32 (19.8%)	78 (20%)
Patients with diabetes are allowed to consume dried mulberry in any amount.	122 (53%)	79 (49.1%)	201 (51.5%)
Patients with diabetes can consume any amount of fruit.	81 (35.4%)	62 (38.5%)	143 (36.6%)
Patients with diabetes are allowed to consume raisins in any amount.	77 (33.6%)	50 (31.1%)	127 (32.5%)
Patients with diabetes are allowed to consume dried figs in any amount.	64 (27.9%)	49 (30.4%)	113 (28.9%)
Patients with diabetes are allowed to consume date in any amount.	55 (24%)	39 (24.2%)	94 (24.1%)
Patients with diabetes can consume honey in any amount because it has natural sugar.	46 (20.1%)	36 (22.4%)	82 (21.02%)
Patients with diabetes can consume juices in any amount because fruit sugar is natural.	24 (10.5%)	25 (15.5%)	49 (12.5%)
2‐3. Oils and Fats			
Only solid oils are fattening.	143 (62.4%)	83 (51.6%)	302 (77.4%)
Butter and cream can be used in any amount because they are dairy products.	51 (22.3%)	35 (21.7%)	86 (22%)
Using animal oils is beneficial for diabetes.	40 (17.4%)	31 (19.3%)	71 (18.2%)
2‐4. Water intake			
Water intake should be decreased when the urine amount increases.	38 (16.6%)	31 (19.3%)	69(17.6%)
**Field 3 ‐ Diabetes management and follow‐up misconceptions**			
3‐1. Management			
Herbal remedies can be used instead of medical treatment.	46 (20%)	35 (21.7%)	81 (20.7%)
Medication should be taken only when the patient feel that blood sugar is high.	9 (3.9%)	5 (3.1%)	14 (3.5%)
Medication should be taken only after a sweet meal.	6 (2.6%)	4 (2.5%)	10 (2.5%)
Medication should be taken whenever I remember.			4 (1%)
3‐2. Best laboratory evaluation interval			
As prescribed			280 (72%)
Whenever possible			81 (21%)
People who are currently undergoing medical treatment do not need to take follow‐up laboratory tests.	14 (6.1%)	15 (9.3%)	29 (7.4%)
3‐3. Treatment continuation			
As newly prescribed after the latest doctor's note.			233 (60%)
As prescribed previously without getting a new prescription.			27 (7%)
Medication should be decreased if laboratory follow‐up tests are normal.	13 (5.7%)	14 (8.7%)	27 (14%)
Medication should be stopped if laboratory follow‐up tests are normal.	4 (1.7%)	4 (2.5%)	8 (2.05%)
3‐4. Medication on the day of laboratory evaluations			
Medication should be taken as prescribed on the morning of routine blood sugar evaluation.	78 (34.1%)	44 (27.3%)	122 (31.2%)
Medication should be discontinued the day before the routine blood sugar evaluation.	16 (7%)	18 (11.2%)	34 (8.7%)
Insulin must be injected as prescribed on the morning of the routine blood sugar evaluation.	31 (13.5%)	18 (11.2%)	49 (12.5%)
Insulin must be discontinued the day before the routine blood sugar evaluation.	12 (5.2%)	11 (6.8%)	23 (5.8%)
3‐5. Concerns about the potential requirement for future insulin treatment			
Injection pain			
Inconvenience of injection			
The risk of hypoglycemia			
Nonavailability			
The risk of dependency			
Economic issues			
**Field 4 ‐Misconceptions regarding diabetes complications and social stigma**			
Complications occur even with standard management.	42 (18.3%)	35 (21%)	77 (19.7%)
It is better not to marry an individual with diabetes.			
Should not marry an individual with diabetes.			

### 3.2 Pathogenesis Misconceptions

Several misconceptions about the nature, causes, and pathogenesis of diabetes were identified. As shown in Table 2, the most common, reported by 177 participants (45.3%), was that diabetes is only a hereditary disease. This was followed by the misconception that only obese individuals can develop diabetes, reported by 167 participants (42.8%).

### 3.3 Nutritional and Lifestyle Misconceptions

Questions related to nutritional information showed that patients had a fairly appropriate mindset. However, the most common misconception in this category was that only solid oils are fattening, reported by 302 participants (77.4%) (Table 2). Furthermore, a pattern of foods was seen that patients believed must be discontinued entirely in diabetes; the mentioned ones were pasta [125 (32%)], potato [88 (22.5%)], rice [78 (20%)], and bread [78 (20%)]. Additionally, misconceptions about products that can be consumed in any amount during diabetes showed that 201 (51.5%) chose dried white mulberries, 143 (36.6%) chose fruits, 133 (34%) chose meat products, 127 (32.5%) chose raisins, 113 (28.9%) chose dried figs, 94 (24.1%) chose dates, 82 (21%) chose honey, and 49 (12.5%) chose juices. Moreover, 86 (22%) patients mentioned that when faced with increased levels of urination, they would increase their fluid intake, 69 (17.6%) said that they would decrease their water intake, while 235 (60%) correctly indicated that they do not change their drinking pattern.

### 3.4 Diabetes Management Misconceptions

In the field of DM management, the most common misconception was that herbal remedies can be used instead of medical treatment, reported by 81 participants (20.7%) as indicated in Table 2. When asked whether they have tried any home remedies or herbal medicine, 125 (32%) answered positively, citing various selections. The most common ones were Urtica, Eryngium, Okra, Cinnamon, and Fenugreek. When asked about the timing of medication use, 362 (93%) answered “as prescribed.” For the best interval of laboratory evaluation, 280 (72%) chose “as prescribed,” while 81 (21%) selected “whenever possible” due to difficulty or lack of time. Regarding continuation of treatment when blood sugar is within normal ranges, 233 (60%) chose “as newly prescribed after the latest doctor's note,” and 122 (31%) said “as prescribed previously without getting a new prescription.” For laboratory evaluations, a misconception was identified in 122 (31.2%) of participants who believed they should take their medications on the morning of testing, while 34 (8.7%) said they should stop medications the day before. Among insulin users, 49 (12.5%) believed insulin should be used on the morning of testing, and 23 (5.8%) believed it should be discontinued the day before. Around 190 (48.7%) of participants expressed concerns about the potential requirement for future insulin treatment. The top 5 concerns were injection pain (76/190), inconvenience (41/190), risk of hypoglycemia (34/190), nonavailability (33/190), and fear of dependency (28/190). Men were significantly more concerned about economic issues and market availability of insulin compared to women (83.3% vs. 57.6%, respectively).

### 3.5 Misconceptions Regarding Diabetes Complications and Social Stigma

The most frequent misconception in this category was that complications occur even with standard management, reported by 77 participants (19.7%) (Table 2). This reflects a sense of hopelessness about disease outcomes. Additionally, approximately one‐quarter of the patients believed that it is better not to [77 (19.7%)] or should not [19 (4.9%)] marry an individual with diabetes.

### 3.6 Gender‐ and Age‐Specific Differentials in Misconceptions

Table 2 presents sex‐stratified frequencies of misconceptions. Notably, the incorrect assumption that only obese individuals can develop diabetes was significantly more common among women than men (88/167 F vs. 79/167 M) (*χ* ^2^ = 4.371, df = 1, *p* = 0.037, Φ = 0.16), suggesting a gender‐specific misunderstanding of diabetes etiology.

Age patterns were also evident. Since the number of young adults was small, to compare the distribution of misconceptions in age groups, the young adults and adults were merged into one group, named “adult.” There were significant age difference patterns in particular questions, for example, the frequency of the misconception “Patients with diabetes are allowed to consume raisins in any amount,” in adult, middle‐age, and old‐age groups was equal to 13 (17.8%), 52 (32.7%), and 62 (39.2%), respectively (*χ* ^2^ = 10.447, df = 2, *p* = 0.005, Cramer's *V* = 0.2). Regarding the misconception “Butter and cream can be used in any amount because they are dairy products,” the frequency in the three groups was equal to 9 (12.3%), 28 (17.6%), and 49 (31%), respectively (*χ* ^2^ = 13.221, df = 2, *p* = 0.001, Cramer's *V* = 0.28). The frequency of misconception in “Complications occur even with standard management” in the order of increasing age group was equal to 7 (9.6%), 24 (15.1%), and 46 (29.1%) (*χ* ^2^ = 15.675, df = 2, *p* = 0.001, Cramer's *V* = 0.32). This trend indicates that certain misconceptions increase with age, showing a medium to large effect size.

### 3.7 Correct Knowledge

Due to the nature of the questionnaire design, there were also results regarding the correct perception and beliefs of patients about diabetes, which are as follows:

In total, 188 (48%) checked that an unhealthy lifestyle is the leading cause of diabetes. A total of 320 (82%) believed they must consume three main meals, and 129 (33%) checked that they have to consume three snacks daily.
Regarding patients' general knowledge about diabetes complications, some of the most popular choices were retinopathy by 238 (61%), nephropathy by 204 (52.3%), ischemic heart disease by 82 (21%), foot ulcers by 75 (19.2%), neuropathy by 54 (13.8%), and amputation by 49 (12.6%).

## 4 Discussions

Our study revealed many misconceptions regarding DM among people suffering from this disease. The top 5 false beliefs based on our findings include “Only solid oils are fattening,” “Patients with diabetes are allowed to consume dried mulberry unlimited,” “Diabetes is only a hereditary disease,” “Only obese people can get diabetes,” and “Patients with diabetes can consume any amount of fruit.” To our knowledge, no previous studies conducted in Iran have reported such a comprehensive overview of diabetes‐related misconceptions. This approach enabled us to capture a broader range of misconceptions, contributing to a more complete and nuanced understanding of patient beliefs.

Misinformation and misconceptions play a detrimental role in disease outcomes. An educated patient with a knowledgeable mindset is considerably less prone to the burdens of DM [17]. Different areas of knowledge, such as nutrition, treatment, follow‐up management, complications, and lifestyle, need to be considered when faced with a patient with diabetes during initial and follow‐up visits [18].

Even though the overuse of all types of dietary fat is fattening, a common misconception was that only solid oil products (saturated fatty acids) can lead to obesity. More than 25% of the patients mentioned that fruit, meat, raisins, and dried figs could be taken without limitation. Patients could view these products as less harmful because they contain natural sugars. However, they can still raise blood sugar levels and should be consumed with caution. When fruit is dried, the removal of water concentrates its natural sugars and calories into a smaller, more compact form. As a result, dried fruits become particularly rich in both calories and sugars, including glucose and fructose [19]. More than 25% of participants believed that all fruits could be consumed without limitation. However, fruits high in fructose and with a high glycemic index, such as mangoes, bananas, melons, and peaches, should be consumed in moderation [20].

Similarly, some studies have shown that the highest misconception rates are from dietary misinformation. For example, a survey by Zowgar and colleagues showed that, on average, only 37.9% of patients had correct dietary conceptions and questions regarding this matter were the most incorrectly answered [17].

The higher frequency of certain nutritional misconceptions among older individuals may be attributed to younger generations having greater access to accurate information through technology, whereas older adults are more inclined to retain culturally ingrained beliefs.

Two of the wrong beliefs in this study were that diabetes is solely a hereditary condition, meaning there is no risk if no family members are affected, as well as thin people do not develop diabetes. This result has also been revealed in some other studies [9, 21, 22]. When a patient believes that diabetes is exclusively caused by heredity or obesity, it becomes important from two perspectives. First, patients may neglect lifestyle and weight management. Second, they might avoid early screening tests, potentially delaying diagnosis until the complications have already set in. Promoting health awareness and addressing these misconceptions through early education, starting in childhood, can help prevent this. Regarding the cause of diabetes, more women than men believed that only obese individuals were affected, though the effect size was small. This may suggest that women either have less information on the topic or are more influenced by nonmedical sources.

The misconception regarding the etiology of diabetes was higher in women which was in line with other studies [9, 23]. However, there was no gender difference in Sharaf et al.'s study [24].

An interesting finding in our study was the widespread use of herbal medicine among patients with diabetes. Similarly, a 2013 study by Patil and colleagues reported “diabetes can be cured by herbal treatment,” as the most common misconception among diabetic patients (46.6%) [18]. One of the possible reasons is the assumption that herbal medicine has fewer side effects than synthetic drugs, due to its natural source. Although there are many herbal remedies available for the treatment of diabetes, only a few of them have been scientifically and medically evaluated to measure their effectiveness [25]. Additionally, even for herbal agents with evidence‐based antidiabetic effects, their use should be restricted without a doctor's prescription.

Contrary to informal and verbal reports from service providers regarding the use of opium for diabetes management, our study did not identify any mention of this practice among the participants. This could be attributed to the social stigma surrounding opium use, which may have deterred open discussion during interviews. The use of opium as a means to prevent diabetes complications and related health issues remains a controversial subject in certain communities. While some studies suggest that opium may elevate blood glucose and lipid levels, others report no significant association between opium use and diabetes control [26, 27]. Another study indicates that although opium may cause a transient decrease in blood glucose, it does not have a consistent or long‐lasting effect, as reflected by its negligible impact on HbA1c levels [28].

Effective follow‐up care is also a crucial aspect of diabetes management; a total of 103 patients in our study were treated with insulin (with or without oral medication), but only 59.2% of them were aware of the correct timing to withhold insulin on the day of blood sampling. This implies that 40.8% lacked proper knowledge about withholding insulin before blood sampling, which poses a risk of misinterpreting laboratory results. Such errors can lead to improper insulin dose adjustments, resulting in either hypoglycemia or hyperglycemia. Previous evidence from an Indian study also suggests that 52.41% of DM patients are unaware of insulin injection timings, underscoring the importance of patient education on insulin therapy [29]. Furthermore, of the 311 patients on oral medications (with or without insulin therapy), only 54.6% knew the appropriate timing for withholding medication prior to blood sampling. Comprehensive patient education regarding both nutritional and pharmacological preparations before testing is essential to avoid side effects and ensure accurate disease management.

Over 51% of patients expressed concerns about insulin therapy, and 19.7% reported feelings of hopelessness about developing complications despite standard treatment, raising the likelihood of incomplete treatment adherence. It is imperative to address these concerns and explain the benefits of standard therapies in clear, simple language. Healthcare providers should work to identify and correct common misconceptions surrounding diabetes management, especially during patient visits. While a complete education covering all aspects of diabetes care may not always be feasible in terms of time and resources, we recommend that healthcare providers prioritize addressing misconceptions at each visit, focusing on the most common false beliefs, and delivering concise, culturally sensitive education, using easily understandable language. Utilizing family members as allies in education may also enhance understanding and compliance [30].

In Iran's healthcare system, patient education guidelines are in place, but the high patient load often limits the time available for thorough education [31]. Moreover, in Iranian culture, family members and close associates have a significant influence on patients' health beliefs, sometimes outweighing the impact of healthcare providers [32].

Many misconceptions, particularly those related to treatment, are deeply rooted in the culture of the people, including a longstanding interest in herbal remedies [33]. Addressing these deeply ingrained misconceptions poses a considerable challenge for the healthcare system, requiring a multifaceted approach. Currently, various interventions are being implemented in Iran to increase public awareness of health issues, such as putting up banners in main city squares in coordination with the municipality, expert‐led radio and television programs, and educational materials displayed in healthcare centers. However, our findings suggest that these efforts remain insufficient. Successful educational initiatives will require collaboration between multiple sectors, including the Ministry of Health, media outlets, nongovernmental organizations, and local communities. Furthermore, ongoing evaluation and feedback from the target population are critical for refining these educational strategies.

## 5 Limitations

The primary limitation of this study is the use of convenience sampling, as implementing random sampling was not feasible due to the lack of a centralized patient registry and the wide dispersion of healthcare facilities across the region. However, we sought to reduce potential bias through a structured two‐stage approach: we purposively selected a diverse range of healthcare centers to capture variation in patient demographics, socioeconomic status, and care settings; and within each site, we employed consecutive enrollment, proportional site coverage, and varied visit days and times. While the sample is not probabilistic, these steps were taken to enhance representativeness and minimize selection bias.

Another limitation is the lack of representation from diverse ethnic and religious groups, as the majority of Iran's population is Persian and follows Islam. Furthermore, the study did not assess the sources of misconceptions, which could have provided valuable insights into their origins. Additionally, recording HbA1C values for all patients would have enhanced the accuracy and depth of understanding regarding these misconceptions.

Lastly, the questionnaire was developed based on a literature review, which, while thorough, may not capture the full scope of diabetes‐related misconceptions in Iranian communities. We recommend conducting qualitative research in various regions of Iran to gain a more comprehensive understanding of these misconceptions and their contributing factors.

## 6 Conclusion

This study highlights the widespread misconceptions about diabetes among patients, with the potential to negatively impact diabetes management and glycemic control. Common myths, such as the false belief that only obese individuals can develop diabetes or the misconception about insulin use, underline the importance of accurate patient education. Our findings revealed several critical areas where patients' misunderstandings contribute to poor disease management, such as misconceptions about dietary practices, treatment methods, and diabetes complications.

## 7 Recommendation

Media, as a powerful communication tool, can play a pivotal role in disseminating reliable health information to large audiences [34]. Additionally, implementing standardized counseling frameworks can ensure that all patients receive accurate, consistent information, regardless of their background [35]. Educating patients properly equips them to challenge societal and cultural misconceptions, leading to better adherence to treatment plans and healthier lifestyle choices. Furthermore, fostering a well‐informed patient population enhances the relationship between patients and their healthcare providers, ultimately contributing to better long‐term health outcomes. By addressing both the misconceptions and the gaps in patient knowledge through comprehensive educational strategies, we can pave the way for improved diabetes management and a healthier population. In addition, we recommend that the findings of this study be shared with national health policymakers to support the integration of structured educational modules into the existing diabetes care program. These modules should equip healthcare providers with tools to identify and address patient misconceptions more effectively, improving disease management and health outcomes. Further quantitative evaluations could also provide a clearer understanding of the scale of misinformation, helping to prioritize areas for intervention and track changes over time to assess the impact of educational efforts.

## Author Contributions

**Anahita Babak:** conceptualization (lead), methodology (lead), supervision (lead), validation (lead), writing – original draft (equal). **Shiva Rouzbahani:** validation and visualization (equal), writing – original draft (equal), writing – review and editing (lead). **Alireza Safaeian:** formal analysis (lead), writing – original draft (equal). **Farzam Poonaki:** investigation (lead), writing – original draft (lead), corresponding. The authors all participated in the review, drafting, and final approval of the manuscript, endorsed the final version for publication, and committed to taking responsibility for every aspect of the project. They ensured thorough investigation and resolution of any inquiries regarding the accuracy or integrity of the work. All authors have read and approved the final version of the manuscript.

## Ethics Statement

This study has been approved by the Research Ethics Committees of the School of Medicine – Isfahan University of Medical Sciences and adheres to the guidelines outlined in the 1995 Declaration of Helsinki (as revised in Fortaleza, Brazil, October 2013). Ethic Code: IR.MUI.MED.REC.1401.276. Approval date: October 28, 2022.

## Consent

All participants provided informed consent to take part in the study.

## Conflicts of Interest

The authors declare no conflicts of interest.

## Transparency Statement

The lead author Anahita Babak affirms that this manuscript is an honest, accurate, and transparent account of the study being reported; that no important aspects of the study have been omitted; and that any discrepancies from the study as planned (and, if relevant, registered) have been explained.

## Data Availability

The data that support the findings of this study are available from the corresponding author upon reasonable request. Farzam Poonaki had full access to all of the data in this study and takes complete responsibility for the integrity of the data and the accuracy of the data analysis.
